# Effects of Lactate on Improving Cognitive Function and Survival Rate in a Mouse Model of Post-Sepsis Cognitive Impairment

**DOI:** 10.62641/aep.v54i2.2165

**Published:** 2026-04-15

**Authors:** Jinyong Huang, Haiyong Liu, Yongwei Wu, Xiaochun Yuan, Yongtao Gao

**Affiliations:** ^1^Department of Anesthesiology, Affiliated Hospital of Nantong University, 226001 Nantong, Jiangsu, China; ^2^Medical College of Nantong University, 226001 Nantong, Jiangsu, China; ^3^Department of Anesthesiology, Yancheng City Dafeng People’s Hospital, 224100 Yancheng, Jiangsu, China; ^4^Department of Critical Care Medicine, Yancheng City Dafeng People’s Hospital, 224100 Yancheng, Jiangsu, China

**Keywords:** post-sepsis cognitive impairment, lactate, energy metabolism, oxidative stress, HMGB1/RAGE axis, neuroprotection

## Abstract

**Background:**

Post-sepsis cognitive impairment (PSCI) represents a prevalent complication observed in survivors of sepsis, manifesting as neurocognitive deficits, including memory impairment and attentional deficits. The precise mechanisms contributing to PSCI are inadequately understood, and there is a notable absence of effective clinical interventions. Lactate, previously considered as a metabolic byproduct, has recently been recognized as a potential energy source and signaling molecule involved in various physiological and pathological processes that are intricately linked to clinical outcomes of sepsis. This study aimed to explore the neuroprotective effects of lactate on PSCI and to elucidate the underlying mechanisms.

**Methods:**

Sixty male C57BL/6 mice were randomly assigned to three groups: control, sepsis-induced cognitive impairment, and lactate treatment groups. Sepsis-associated cognitive impairment was induced by cecal ligation and puncture. Lactate was administered via daily intraperitoneal injection (1 mol/L, 10 μL/g) for 10 consecutive days beginning 24 hours after surgery, while control groups received saline. Survival and body weight were monitored for 20 days. Cognitive performance was evaluated between days 10 and 15 using established behavioral paradigms. Brain tissue was subsequently collected for histological and molecular analyses.

**Results:**

Lactate administration significantly improved survival rates, promoted body weight gain, and enhanced cognitive performance. Additionally, lactate inhibited the activation of the high mobility group box 1 (HMGB1)/receptor for advanced glycation end products (RAGE) axis, reduced neuronal apoptosis and damage, suppressed glial cell activation, reduced Ca^2+^ levels, and modulated inflammatory cytokine production. Mechanistically, lactate upregulated the expression of glucose transporter 1 (GLUT1) in brain tissue. This facilitated glucose utilization and diminished pyruvate accumulation, potentially influencing energy metabolism. Concurrently, lactate also reduced Malondialdehyde (MDA) levels and elevated Superoxide Dismutase (SOD) activity to mitigate oxidative damage.

**Conclusions:**

Lactate emerges as a promising neuroprotective agent against PSCI potentially influencing cerebral energy metabolism, alleviating oxidative stress, and inhibiting neuroinflammation and neuronal damage.

## Introduction 

Sepsis is a major challenge in intensive care medicine globally, affecting nearly 50 million people each year and leading to over 11 million deaths [1, 2]. In addition to high mortality, sepsis survivors often face long-term complications, among which post-sepsis cognitive impairment (PSCI) is one of the most common and prominent [3, 4, 5, 6, 7, 8]. PSCI refers to the persistent cognitive dysfunction that occurs after sepsis. Its clinical manifestations include memory decline, attention deficits, executive dysfunction, and slowed processing speed, severely affecting long-term quality of life and imposing a heavy burden on families and society [9, 10, 11].

From a psychiatric perspective, PSCI falls into the category of acquired cognitive disorders. Its core pathological feature is progressive or persistent impairment across cognitive domains, caused by sepsis-induced systemic inflammation and subsequent brain injury [12]. PSCI not only overlaps clinically with mild cognitive impairment and major neurocognitive disorder but also possesses distinct characteristics of post-infectious onset. Clinically, patients with PSCI often present with concurrent emotional and behavioral abnormalities such as anxiety, depression, and apathy [12], which further exacerbate the impairment of social functioning and activities of daily living, highlighting the important clinical significance of PSCI in the field of psychiatry. It is noteworthy that sepsis-induced cognitive impairment is not a transient neurological complication; instead, it is more likely to progress to long-term neurocognitive dysfunction [9], thus emerging as a crucial link between critical illness sequelae and psychiatric sequelae. Therefore, in-depth exploration of the pathological mechanisms and intervention strategies of PSCI is of great value for improving the long-term prognosis of sepsis survivors.

Currently, the pathophysiological mechanisms of PSCI are complex and not fully understood. It has been associated with various factors such as neurotransmitter imbalance, neuroinflammation, neuronal apoptosis, oxidative stress, and disorders of energy metabolism [9, 10, 13, 14, 15]. Therapeutic strategies targeting these pathophysiological mechanisms have shown limited effectiveness, highlighting the need to explore new pathological targets and potential therapeutic strategies for PSCI.

The role of lactate in sepsis is controversial. On one hand, as an end-product of glycolysis, elevated serum lactate levels are a crucial predictor of disease severity and poor prognosis in sepsis [16, 17, 18]. Consequently, lactate has long been considered a metabolic waste product and a danger signal [19, 20, 21]. On the other hand, modern research has overturned this traditional view, demonstrating that lactate serves as a key energy substrate for multiple organs, including the brain, and is an important metabolic regulator and signaling molecule [22, 23, 24, 25]. This striking duality complicates the specific role of lactate in PSCI. Existing literature reflects this controversy. Some studies suggest that lactate may exacerbate sepsis-associated brain injury by enhancing inflammatory responses [26, 27]. However, in other conditions associated with cognitive impairment, such as Alzheimer’s disease and traumatic brain injury, maintaining lactate metabolic homeostasis and supplementing exogenous lactate are critical for preserving neurological function [22, 23]. In our preliminary experiments, an intriguing phenomenon was observed: exogenous lactate supplementation appeared to improve cognitive function in septic mice. Based on this, we hypothesize that exogenous lactate supplementation may ameliorate PSCI.

Therefore, this study focuses on the key role of energy metabolism, using *in vivo* and *in vitro* experiments to explore the protective effects of lactate on cognitive dysfunction following sepsis and its underlying molecular mechanisms. These findings provide experimental support for the clinical prevention and treatment of PSCI.

## Materials and Methods

### Animal Preparation

Sixty male C57BL/6 mice (8 weeks old, weighing 22 g) were purchased from Aniphe Biolaboratory Inc. (Jiangsu, China) in June 2024 and acclimated for 1 week in a specific pathogen-free environment. The mice were housed under controlled conditions with a temperature of 20 to 25 °C, humidity of 50% to 70%, free access to food and water, and a 12-hour dark/light cycle. All animal experiments were approved by the Ethics Committee of Aniphe Biolaboratory Inc. (Jiangsu, China) (Ethics approval number: JSAB24026M) in accordance with the Animal Research: Reporting of *In Vivo* Experiments (ARRIVE) guidelines and adhered to the basic principle of minimizing distress and harm to the animals.

### Group Setting

All experimental mice were randomly divided into three groups: Control group (n = 10), PSCI group (n = 25), and Lactate group (n = 25). The PSCI model was established using cecal ligation and puncture (CLP) [28, 29, 30, 31, 32]. Mice were anesthetized by inhalation of 3% isoflurane (DM-M9462, DUMABIO, Shanghai, China), placed in a supine position, and secured on a foam surgical table. The abdominal fur was trimmed, depilated, and disinfected, then a midline abdominal incision of about 2.0 cm was made to expose and exteriorize the cecum. The cecum was ligated distal to the ileocecal valve and punctured twice with a 21-G needle. After gently squeezing the cecum to expel a small amount of feces, it was repositioned inside the abdominal cavity, and the skin was sutured layer by layer. Mice in the Control group underwent a sham operation without CLP. For the Lactate group, mice received a daily intraperitoneal injection of lactate-saline solution (1 mol/L, 10 µL/g; 71718, Sigma, Munich, Germany) for 10 consecutive days, starting from day 1 post-CLP [30, 31, 32]. As vehicle controls, mice in the PSCI and control groups were administered an equivalent volume of saline at the same time points. Survival rates and body weight changes were recorded within 20 days post-surgery. Behavioral tests (Morris water maze, Y-maze, and novel object recognition test) were performed on days 10 to 15. On day 20 after surgery, mice were euthanized via cervical dislocation, and their brains were harvested for Hematoxylin-eosin staining and Nissl staining.

### Cells and Treatment 

Mouse cerebellar astrocytes (C8-D1A) and mouse microglial cells (N9) were obtained from the Cell Bank of Chinese Academy of Sciences (Shanghai, China) and cultured in Iscove’s Modified Dulbecco’s Medium (Gibco, Carlsbad, CA, USA) containing 5% fetal bovine serum, 2 mmol/L glutamine, 100 U/mL penicillin, 100 µg/mL streptomycin, and 50 µM 2-mercaptoethanol (F2442, Sigma, Munich, Germany). Cells were maintained in a humidified incubator at 37 ℃ with 5% CO_2_. When cell confluence rate reached approximately 80%, cells were pretreated with lipopolysaccharide (LPS, 1 µg/mL, YT1319, Ittabio, Beijing, China) for 24 hours to induce an inflammatory model, followed by treatment with lactate (0.9 mg/mL) for 24 hours. Cells were grouped according to different pretreatment methods: a control group (no pretreatment), an LPS group (LPS pretreatment), and a lactate group (lactate added after LPS pretreatment) [30, 31, 32].

The C8-D1A and N9 cell lines were cultured to 80% confluence and then pretreated with LPS (1 µg/mL) for 2 hours to establish an inflammatory model, followed by division into three groups: small interfering RNA–negative control (si-NC) group (LPS pretreatment); si-NC lactate group (0.9 mg/mL lactate administered after LPS pretreatment + si-NC); and si-lactate dehydrogenase A (LDHA) group (LPS pretreatment + si-LDHA).

### Morris Water Maze (MWM)

On day 1 of adaptive training, mice were placed in a pool to swim freely for 10 minutes to acclimate to the environment. From days 2 to 5, during the spatial navigation experiment, the pool was evenly divided into four quadrants, with a platform placed at the center of one quadrant, 1 cm below the water surface. Mice were introduced into the water from different quadrants, each facing the pool wall. Escape latency, defined as the time required to locate the platform within 120 seconds, was recorded. Mice that failed to reach the escape platform were manually guided to the platform to rest for 20 seconds, and their escape latency was recorded as 120 seconds. Subsequently, in the probe trial on day 6, the platform was removed, and mice were introduced into the water from the quadrant opposite to the original platform location. The number of platform crossings and their total swimming distance within 120 seconds were recorded.

### Y-maze

Mice were placed into a Y-shaped maze (40172, Ugo Basile, Varese, Italy) with three arms angled at 120°, each arm measuring 30 cm long, 5 cm wide, and 15 cm high. Mice were allowed to explore freely for 8 minutes. The frequency of arm entries and the number of spontaneous alternations percentage (SAP) were recorded. SAP behavior was defined as continuous entries into three different arms. The percentage of spontaneous alternations was calculated using the formula: SAP % = (actual alternations)/(total number of arm entries – 2) × 100%.

### Novel Object Recognition (NOR)

On day 1 (adaptation), mice were placed individually in a 50 × 50 × 50 cm box for free movement for 10 minutes. On day 2 (acquisition), two identical objects were placed in the arena, and mice were allowed to explore for 10 minutes. On day 3 (test), mice were placed in the same box to explore for 10 minutes; immediately before this phase, one new object replaced one of the familiar objects. The time spent exploring the objects during both the acquisition and test phases was recorded. Discrimination Ratio = (time spent exploring the new object)/(time spent exploring the new object + time spent exploring the familiar object) × 100%.

### Sample Collection 

Twenty days after surgery, mice were euthanized for sample collection. For protein and metabolic parameter analysis, a subset of mice was euthanized by cervical dislocation under isoflurane anesthesia. Their brains were rapidly harvested, and one part was stored at –80 °C. For histological analysis, another subset of mice was deeply anesthetized and transcardially perfused with 100 mL of normal saline to flush the blood, followed by perfusion with 4% paraformaldehyde pre-cooled to 4 °C for tissue fixation. The perfused brain tissue was then collected, immersed in 4% paraformaldehyde, dehydrated in graded ethanol, cleared with xylene, and embedded in paraffin. Sections of 5 µm thickness were prepared for further analysis.

### Measurement of Glucose, Pyruvic Acid, Malondialdehyde (MDA), Superoxide Dismutase (SOD)

Brain tissue and cell homogenates were centrifuged at 12,000 ×g for 5 minutes. The supernatant was collected, and the concentrations of glucose (R21650, Orileaf, Shanghai, China), pyruvate (E-BC-K130-M, Elabscience, Wuhan, China), and MDA (BC0020, Solarbio, Beijing, China), as well as the activity of SOD (50104ES60, Yeasen, Shanghai, China), were measured according to the instructions provided with the commercial kits. Absorbance was measured using a microplate reader (Thermo Multiskan FC, ilmington, DE, USA) at 510 nm (glucose), 570 nm (pyruvate), 532 nm (MDA), and 450 nm (SOD).

### Hematoxylin and Eosin (H&E) Staining

Paraffin sections were baked, deparaffinized in xylene, and rehydrated in a graded ethanol series, followed by hematoxylin staining for 5 minutes to stain the nuclei, differentiation using hydrochloric acid in ethanol for 30 seconds, and eosin staining for 3 minutes to stain the cytoplasm (60524ES60, Yeasen, Shanghai, China). After dehydration and clearing, sections were sealed with neutral resin, and the morphology of neurons and the extent of inflammatory infiltration in the hippocampal region of the mouse brain was observed under a light microscope (Olympus, Tokyo, Japan).

### Nissl Staining

After deparaffinization and rehydration, sections were stained with 0.1% toluidine blue solution at 37 °C for 30 minutes (60531ES50, Yeasen, Shanghai, China). Following differentiation, sections were dehydrated, became transparent, and were then sealed with a coverslip. The morphology and distribution of Nissl bodies in hippocampal neurons were subsequently observed.

### Immunofluorescence Staining 

Cells were seeded onto sterile coverslips at a density of 5 × 10^4^/cm^2^. The cells were first fixed with 4% paraformaldehyde for 15 to 20 minutes at room temperature, followed by permeabilization with 0.1% Triton X-100 for 10 minutes. The coverslips were then blocked with 5% bovine serum albumin for 1 hour. Primary antibodies against ionized calcium binding adaptor molecule 1 (IBA1, 1:500, AB312913, Abcam, Cambridge, UK) and glial fibrillary acidic protein (GFAP, 1:1000, AB194324, Abcam, Cambridge, UK) were applied and incubated overnight at 4 ℃. After washing, fluorescent secondary antibodies (1:500, A21208, Thermo Fisher, Wilmington, DE, USA) were incubated at room temperature for 1 h. Subsequently, the cells were stained with 4^′^,6-diamidino-2-phenylindole (DAPI, Thermo Fisher Scientific, Waltham, MA, USA) for 5 minutes. Finally, an anti-fade reagent was applied before observation under a fluorescence microscope (Olympus, Tokyo, Japan).

### Flow Cytometry 

Cells were seeded in 6-well plates at a concentration of 5 × 10^5^ cells/mL and cultured for 24 hours until 80% confluence. Cells were then digested with 0.25% trypsin for 1 minutes, and the trypsin digestion was stopped by adding serum-containing medium. After centrifugation at 1000 rpm for 5 minutes, cells were incubated with 2 to 5 µmol/L Fluo-4 AM in the presence of 0.02% Pluronic F-127 for 30 minutes. Following incubation, the cells were washed with Hank’s Balanced Salt Solution and incubated at 37 °C for 15 minutes in the dark. Finally, the cells were analyzed by flow cytometry (E-CK-A211, Elabscience, Wuhan, China) using excitation/emission wavelengths of 488/530 nm.

### Enzyme-Linked Immunosorbent Assay (ELISA)

The cell culture supernatant was collected and centrifuged (2000 rpm, 20 minutes) to remove debris. The levels of interleukin-1 beta (IL-1β; SEKF-0025), interleukin-6 (IL-6; SEKF-0140), tumor necrosis factor alpha (TNF-α; SEKF-0145), interleukin-10 (IL-10; SEKR-0006), interleukin-4 (IL-4; SEKRT-0034), and transforming growth factor beta (TGF-β; SEKR-0013) were measured using an ELISA kit (Solarbio, Beijing, China). The absorbance at 450 nm was measured using a microplate reader (Thermo Multiskan FC, Wilmington, DE, USA), and cytokine concentrations were calculated based on standard curves.

### Determination of Reactive Oxygen Species (ROS)

Cells were seeded into 6-well plates. After the cells reached the appropriate density, serum-free culture medium containing 10 µmol/L 2^′^,7^′^-dichlorodihydrofluorescein diacetate was added (50101ES01, Yeasen, Shanghai, China). The cells were then incubated in the dark at 37 °C for 30 minutes. Subsequently, cells were washed three times with pre-warmed phosphate-buffered saline to remove any unincorporated probes. The levels of intracellular ROS were determined using an Olympus IX53 fluorescence microscope (Olympus, Tokyo, Japan).

### Western Blotting 

Radioimmunoprecipitation assay (RIPA) lysis buffer (20101ES60, Yeasen, Shanghai, China) was used to extract total protein from tissues and cells, and the protein concentration was determined by the Bicinchoninic Acid (BCA) method (Yeasen, Shanghai, China). The protein samples were separated on a 10% sodium dodecyl sulphate-polyacrylamide gel electrophoresis (SDS-PAGE, Bio-Rad Laboratories, Munich, Germany) and then transferred to a polyvinylidene fluoride membrane (Millipore, Billerica, MA, USA). The membrane was blocked with 5% skimmed milk for 1 hour. The primary antibody was added and incubated at 4 ℃ overnight. Subsequently, the membrane was incubated with the secondary antibody (1:500 dilution), at room temperature for 1.5 hours. Protein bands were detected using enhanced chemiluminescence reagents (Yeasen, Shanghai, China), and band intensities were analyzed with ImageJ software (Version 1.53, NIH, Bethesda, MD, USA). The primary antibodies involved in the article are demonstrated in Table 1.

**Table 1.** **Antibody information**.

Antibody name	Catalog number	Company name	Dilution ratio
Anti-Glucose transporter type 1 (GLUT1)	AB22604	Abcam	1:1000
Anti- advanced glycation end-products (RAGE)	AB37647	Abcam	1:1000
Anti- B-cell lymphoma 2 (Bcl-2)	AB194583	Abcam	1:1000
Anti- high mobility group box 1 (HMGB1)	AB227526	Abcam	1:2000
Anti- BCL-2 Associated X (Bax)	AB182733	Abcam	1:1000
Anti-cleaved caspase-3	AB214430	Abcam	1:500
Anti-neuron-specific enolase (NSE)	AB180943	Abcam	1:2000
Anti- CSF S100 calcium-binding protein beta (S100-β)	AB52642	Abcam	1:1000
Anti-GluN2B	HC330013	abinscience	1:500
Anti-GluA1	RHE34601	AntibodySystem	1:1000
Anti-D1R	AB10598308	Proteintech	1:2000
Anti-D2R	ER1907-70	Hua An Biotechnology Company	1:500
Anti- ionized calcium binding adaptor molecule 1 (IBA1)	AB178846	Abcam	1:1000
Anti-glial fibrillary acidic protein (GFAP)	AB68428	Abcam	1:2000
Anti-LDHA	RHB85004	AntibodySystem	1:1000
Anti-glyceraldehyde-3-phosphate dehydrogenase (GAPDH)	AB9485	Abcam	1:5000

### Quantitative RT-PCR Assays

Total RNA was extracted from C8-D1A and N9 cells using an RNA isolation kit purchased from Wuhan Servicebio Technology Co., Ltd. (Bes3018, Wuhan, China). The isolated RNA was reverse-transcribed into complementary DNA (cDNA) with a reverse transcription kit supplied by Nanjing Vazyme Biotech Co., Ltd. (R021-01, Nanjing, China). For real-time quantitative PCR (qPCR), the amplification system was prepared by mixing cDNA templates, specific primers, and SYBR Green Premix (11198ES08, Yeasen, Shanghai, China), followed by detection on a qPCR instrument (Thermo Fisher Scientific, MA, USA). The mRNA expression levels of glyceraldehyde-3-phosphate dehydrogenase (GAPDH) and LDHA were quantified.

The primer sequences used in this study are as follows:

LDHA:

5^′^-ATCTTGACCTACGTGGCTTGGA-3^′^ (forward)

5^′^-CCATACAGGCACACTGGAATCTC-3^′^ (reverse).

GAPDH:

5^′^-ATTGTTGCCATCAATGACCC-3^′^ (forward)

5^′^-AGTAGAGGCAGGGATGATGT-3^′^ (reverse).

All primers were synthesized by GenScript Biotech Corporation (Piscataway, NJ, USA).

### CCK-8 Assay

Cell Counting Kit-8 (CCK-8) was obtained from Beyotime Biotechnology (C0037, Shanghai, China). C8-D1A and N9 cells were seeded into 96-well plates at a density of 5000 cells per well. After respective treatments, 10 µL of CCK-8 solution was added to each well, and the plates were incubated at 37 ℃ for 2 hours. The absorbance was measured at a wavelength of 450 nm using a microplate reader (Thermo Multiskan FC, Wilmington, DE, USA).

### Statistics

All measurement results were expressed as mean ± standard error of the mean (SEM) and analyzed using SPSS 16.0 software (SPSS Inc., Chicago, IL, USA). The Shapiro‒Wilk test was used to assess the normality of the data. The independent-samples *t-*test was used to compare differences between two groups, and one-way analysis of variance followed by Tukey’s post hoc analysis was used for comparisons among three or more groups. When the data did not satisfy the assumptions of normality, the non-parametric Kruskal‒Wallis test was used instead. Results with *p* < 0.05 were considered statistically significant.

## Results 

### Lactate Improved the Survival Rate of Mice With PSCI and Alleviated Body Weight Loss

**Fig. 1.**
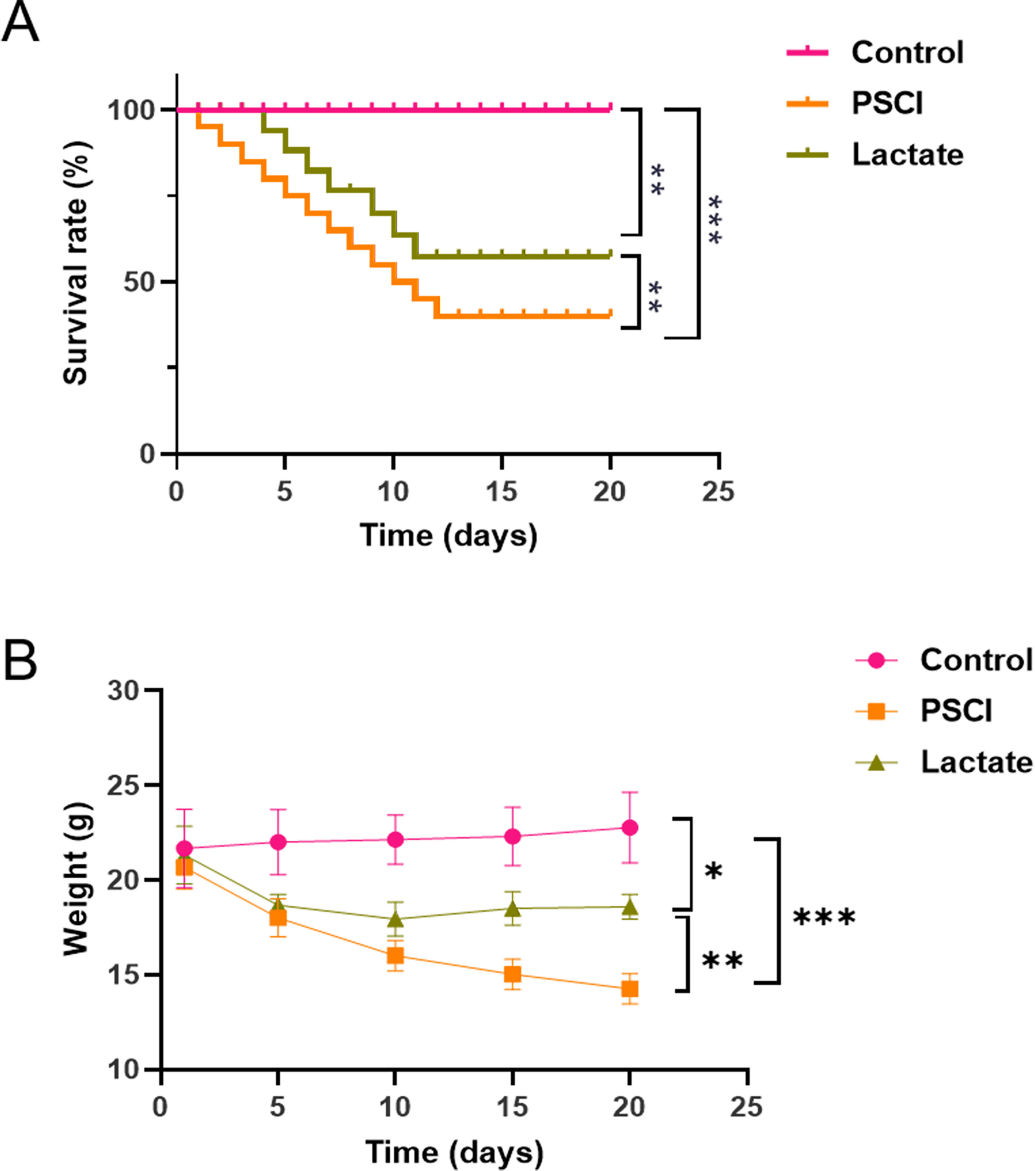
**Lactate improved the survival rate and alleviated weight loss in septic mice**. (A) Kaplan‒Meier survival curves of mice in the sham group (Control), CLP group (PSCI), and CLP + lactate group (Lactate). (B) Weight changes of mice within 20 days post-surgery. Lactate treatment significantly alleviated weight loss in septic mice. Data are presented as the mean ± SEM (n = 10 mice for Control group; n = 25 mice for PSCI and Lactate group). *p < 0.05, **p < 0.01, and ***p < 0.001. CLP, cecal ligation and puncture; PSCI, Post-sepsis cognitive impairment; SEM, standard error of the mean.

Kaplan‒Meier survival analysis showed that, compared with the sham operation group, the survival rates in the CLP group and the lactate group decreased over time. Notably, by postoperative day 20, the survival rate in the lactate group was significantly higher than in the CLP group (Fig. 1A). In addition, although the body weight sharply decreased after CLP-induced sepsis, lactate administration significantly alleviated body weight loss (Fig. 1B), suggesting that lactate improve the overall survival status of septic mice.

### Lactate Enhanced Cognitive Performance in Septic Mice

**Fig. 2.**
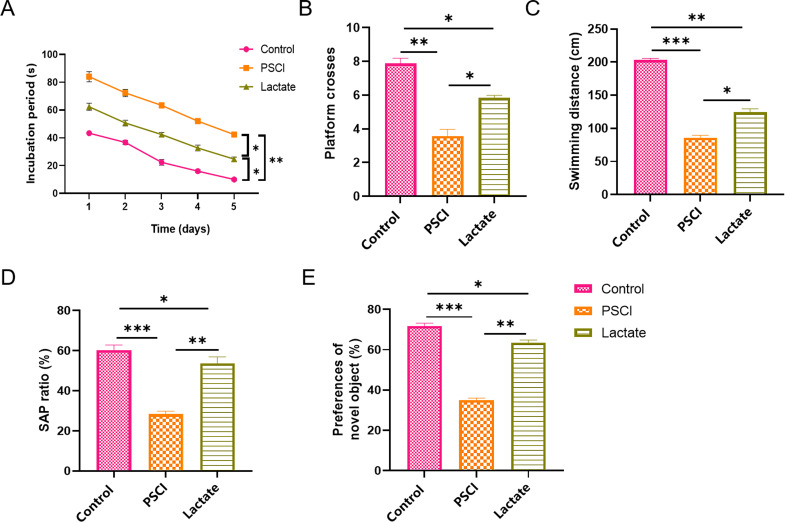
**Lactate alleviated cognitive impairment in septic mice**. (A) Escape latency in the Morris Water Maze (MWM) test. (B) Platform crossing frequency in the MWM test. (C) Total swimming distance in the MWM test. (D) Percentage of spontaneous alternation performance (SAP) in the Y-maze test. (E) Discrimination index in the Novel Object Recognition (NOR) test. Data are presented as the mean ± SEM (n = 5 mice per group). *p < 0.05, **p < 0.01, ***p < 0.001.

In the MWM test, CLP-induced septic mice exhibited a significantly prolonged escape latency, reduced platform crossings, and decreased total swimming distance compared with the control group. These results indicate severe impairment in spatial learning and memory. After lactate treatment, these deficits were significantly improved, specifically manifested as a shortened escape latency, increased platform crossings, and increased total swimming distance (Fig. 2A–C). In the Y-maze test, SAP behavior ratio was reduced in the CLP group, significantly increased after lactate intervention (Fig. 2D). Furthermore, lactate treatment enhanced the discrimination index in CLP-induced septic mice during the novel object recognition test (Fig. 2E).

### Lactate Alleviated Apoptosis, Injury, and Oxidative Stress of Hippocampal Neurons in Septic Mice

**Fig. 3.**
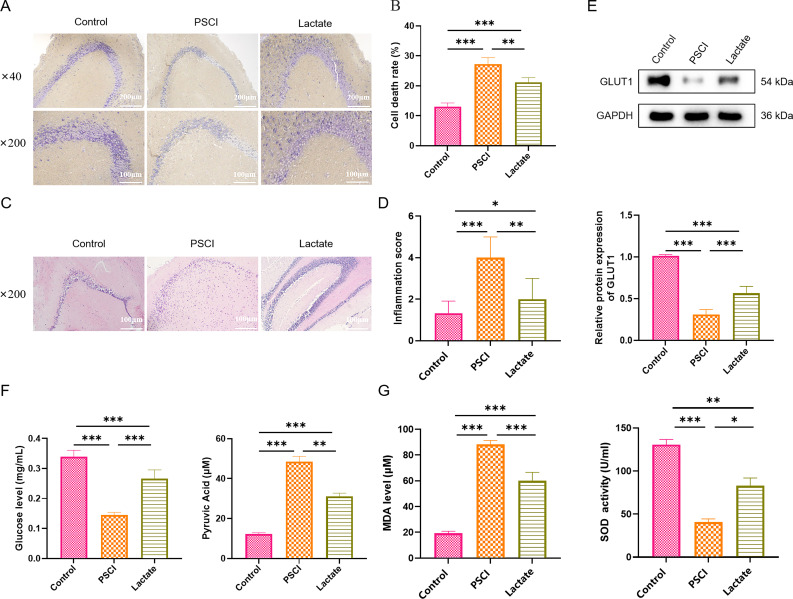
**Lactate alleviated apoptosis, damage, and oxidative stress in hippocampal neurons of septic mice**. (A) Representative images of Nissl staining in hippocampal tissue. (B) Quantification of apoptotic cells. (C) Representative sections of histopathology stained with H&E. (D) Quantification of the inflammation scores based on the H&E staining. (E) Western blot analysis of GLUT1. (F) Quantification of glucose and pyruvate concentrations. (G) ELISA analysis of MDA level and SOD activity. Data are presented as the mean ± SEM (n = 5 mice per group). *p < 0.05, **p < 0.01, ***p < 0.001. Original magnification: ×40, ×200 (A); ×40 (C). Scale bars: 200 µm and 100 µm (A); 100 µm (C). H&E, Hematoxylin and Eosin; GLUT1, glucose transporter 1; ELISA, Enzyme-Linked Immunosorbent Assay; MDA, Malondialdehyde; SOD, Superoxide Dismutase.

Nissl staining showed that the hippocampal neurons in the brain of septic mice were sparsely arranged or even lost, with Nissl bodies sparse or indistinct, nuclei condensed, and cytoplasm shrunken, indicating severe widespread damage. In contrast, the hippocampal neurons of septic mice treated with lactate were densely distributed, with clear nucleoli and abundant Nissl bodies (Fig. 3A). Moreover, lactate significantly reduced the level of apoptotic hippocampal neurons, which was elevated in septic mice (Fig. 3B). H&E staining results showed that CLP-induced neuronal degeneration, necrosis, neuronal loss, glial proliferation, disordered cell arrangement, nuclear condensation, reduced pyramidal neuron size, and infiltration of inflammatory cells were all significantly improved after lactate treatment (Fig. 3C,D). Lactate also upregulated the expression of GLUT1, which were downregulated in CLP-induced septic mice (Fig. 3E). Besides, lactate treatment partially restored the metabolic imbalance in the hippocampus of septic mice, reversing the decrease in glucose concentration and the increase in pyruvate concentration (Fig. 3F). Furthermore, the elevated MDA content was significantly decreased, and the reduced SOD activity was significantly increased in the hippocampus of septic mice after lactate treatment, indicating attenuation of oxidative stress (Fig. 3G).

### Lactate Blocked the HMGB1/RAGE Signalling Pathway, Reduced Apoptosis, and Regulated the Expression of Neurotransmitter Receptors in Septic Mice

**Fig. 4.**
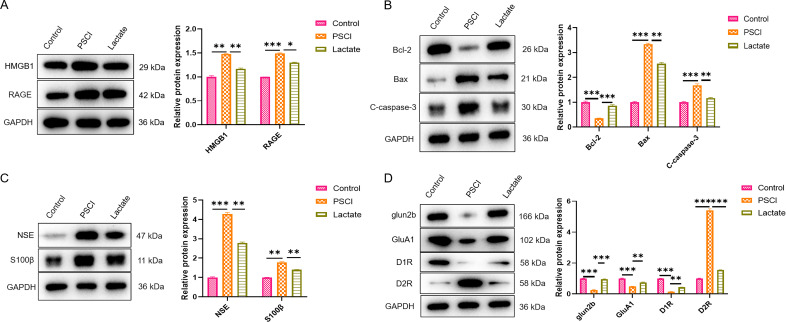
**Lactate blocked the HMGB1/RAGE signalling pathway, reduced apoptosis, and regulated the expression of neurotransmitter receptors**. (A) Western blot analysis of the protein expression levels of HMGB1 and RAGE. (B) Western blot analysis of apoptosis-related proteins (Bcl-2, Bax, cleaved caspase-3). (C) Western blot analysis of brain injury biomarkers (NSE, S100-β). (D) Western blot analysis of neurotransmitter receptors (GluN2B, GluA1, D1R, D2R). Data are presented as the mean ± SEM (n = 5 mice per group). *p < 0.05, **p < 0.01, ***p < 0.001. HMGB1, high mobility group box 1; RAGE, receptor for advanced glycation end products; Bcl-2, B-cell lymphoma 2; Bax, BCL-2 Associated X; NSE, neuron-specific enolase; D1R, dopamine D1 receptor.

Western blot analysis showed that the protein expression levels of high mobility group box 1 (HMGB1) and its receptor RAGE were significantly elevated in the brains of septic mice. Lactate treatment significantly reduced these levels (Fig. 4A). In addition, the expression of the anti-apoptotic protein Bcl-2 was decreased in septic mice, while the pro-apoptotic proteins Bax and cleaved caspase-3 increased; these changes were partially reversed after lactate administration (Fig. 4B). Meanwhile, lactate also reduced the elevated levels of brain injury biomarkers NSE and S100-β in septic mice (Fig. 4C). Furthermore, lactate treatment upregulated the expression of neurotransmitter receptors GluN2B, GluA1, and D1R, while downregulated the expression of D2R in the brain tissue of septic mice (Fig. 4D).

### Lactate Attenuated the Activation of Glial Cells Induced by LPS

**Fig. 5.**
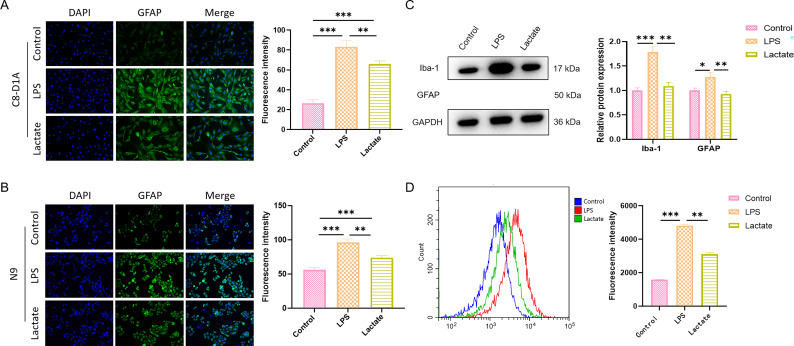
**Lactate attenuated the activation of glial cells induced by LPS**. (A) Immunofluorescence staining for GFAP in C8-D1A cells. (B) Immunofluorescence staining for Iba-1 in N9 cells. (C) Western blotting analysis of GFAP and Iba-1 protein expressions. (D) Flow cytometric assay for intracellular Ca^2+^ concentration. Data are presented as the mean ± SEM (n = 3 for each group). *p < 0.05, **p < 0.01, ***p < 0.001. Scale bars: 50 µm (A,B). LPS, lipopolysaccharide; GFAP, glial fibrillary acidic protein; Iba-1, ionized calcium-binding adaptor molecule 1.

As depicted in Fig. 5A–C, immunofluorescence staining and Western blot analysis indicated that LPS stimulation significantly increased the expression of GFAP in C8-D1A astrocytes. Similarly, LPS significantly increased the expression of Iba-1 in N9 microglial cells. Lactate treatment significantly reduced the expression of these two glial cell activation markers, indicating its inhibitory effect on glial cell activation. Results from flow cytometry analysis showed that the intracellular Ca^2+^ concentration increased in N9 cells stimulated by LPS, while lactate treatment significantly weakened the LPS-induced increase in intracellular Ca^2+^ concentration (Fig. 5D).

### Lactate Exerted Anti-Inflammatory and Antioxidant Effects in LPS-Stimulated N9 Cells

**Fig. 6.**
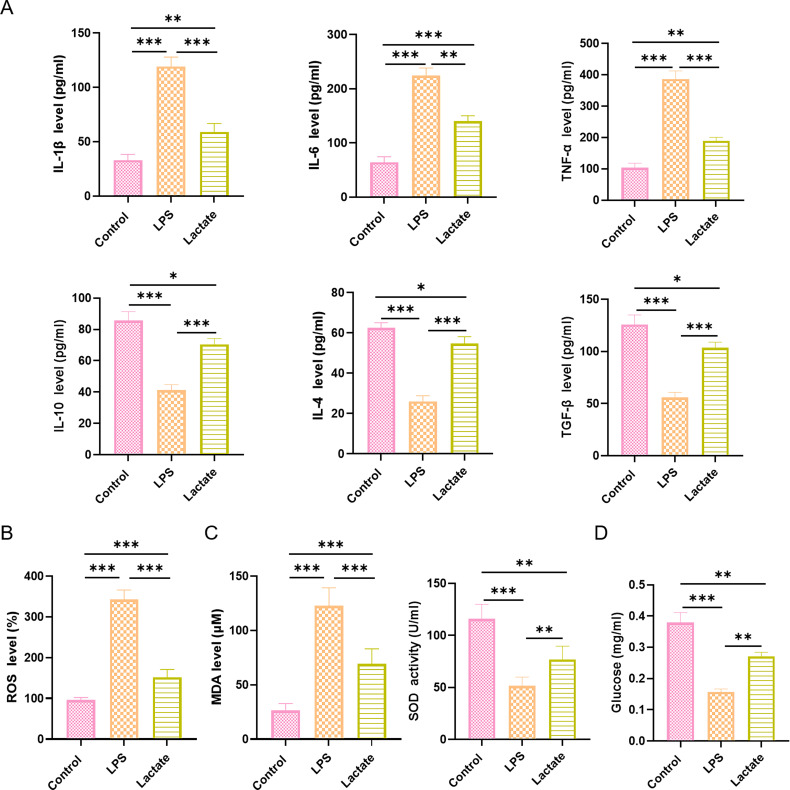
**Lactate exerts anti-inflammatory and antioxidant effects in LPS-stimulated N9 cells**. (A) ELISA analysis of pro-inflammatory cytokines (IL-1β, IL-6, TNF-α) and anti-inflammatory cytokines (IL-10, IL-4, and TGF-β). (B) Detection of intracellular reactive oxygen species (ROS) production. (C) Intracellular MDA level and SOD activity. (D) Quantification of intracellular glucose levels. Data are presented as the mean ± SEM (n = 3 for each group). *p < 0.05, **p < 0.01, ***p < 0.001. IL-1β, interleukin-1 beta; IL-6, interleukin-6; TNF-α, tumor necrosis factor alpha.

The ELISA analysis showed that LPS stimulation significantly increased the concentrations of pro-inflammatory cytokines (IL-1β, IL-6, and TNF-α) in N9 cells, while decreasing the levels of anti-inflammatory cytokines (IL-10, IL-4, and TGF-β). Lactate treatment significantly reversed the altered cytokine levels induced by LPS by reducing the levels of IL-1β, IL-6, and TNF-α and increasing the levels of IL-10, IL-4, and TGF-β (Fig. 6A). Moreover, lactate significantly inhibited the accumulation of ROS induced by LPS in N9 cells (Fig. 6B). Similarly, lactate reduced the content of MDA in LPS-stimulated N9 cells and enhanced the activity of SOD (Fig. 6C). In addition, lactate increased the intracellular glucose levels in LPS-stimulated N9 cells, suggesting a potential role in cellular metabolism regulation (Fig. 6D).

### Knockdown of LDHA Inhibited LPS-Stimulated Glial Cell Viability and Metabolism

**Fig. 7.**
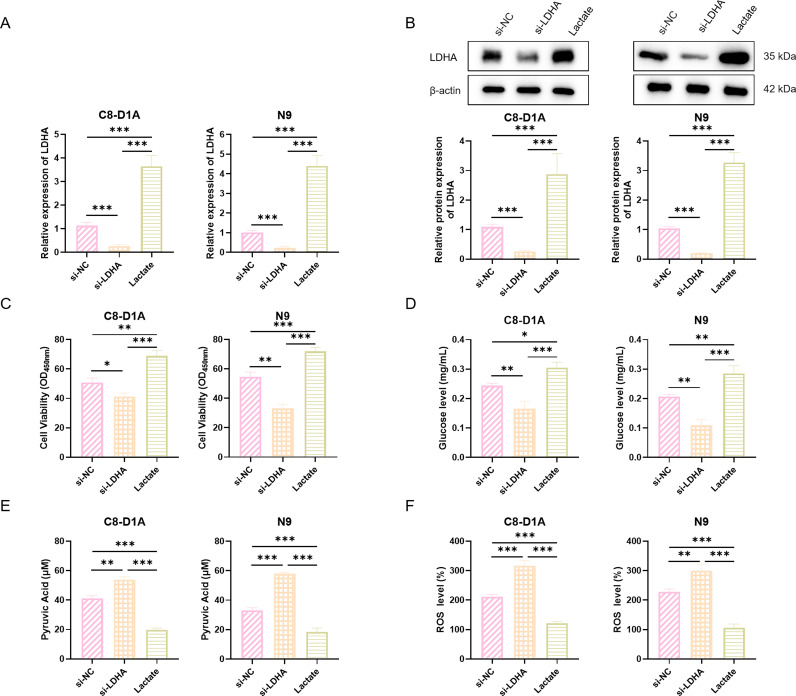
**LDHA knockdown and lactate treatment affect cell viability, metabolism, and ROS levels in C8-D1A and N9 cells**. (A,B) Relative mRNA (A) and protein (B) levels of LDHA in C8-D1A and N9 cells treated with si-NC, si-LDHA, or Lactate. Representative Western blot images and their densitometric quantification are shown in (B). (C) Cell viability of C8-D1A and N9 cells under the same treatments, assessed by CCK-8 assay. (D,E) Intracellular glucose (D) and pyruvate (E) levels in C8-D1A and N9 cells following si-NC, si-LDHA, or Lactate treatment, measured by ELISA. (F) Intracellular ROS levels in C8-D1A and N9 cells assessed by fluorescence assay. Data are presented as the mean ± SEM (n = 3 for each group). *p < 0.05, **p < 0.01, ***p < 0.001. LDHA, lactate dehydrogenase A; ROS, Reactive Oxygen Species; si-NC, small interfering RNA–negative control; CCK-8, Cell Counting Kit-8.

To further elucidate the mechanism of lactate’s action in PSCI, we generated two LDHA knockdown glial cell lines. Firstly, the transfection efficiency of si-LDHA was assessed by qRT-PCR and Western blot. As shown in Fig. 7A,B, we successfully established C8-D1A and N9 cell lines with significantly reduced LDHA expression. Secondly, we compared the viability of C8-D1A and N9 cell lines following LDHA knockdown. CCK-8 assay results indicated that LDHA knockdown significantly decreased the viability of both C8-D1A and N9 cell lines. The Lactate group exhibited significantly higher cell viability compared to the si-NC group in both cell lines (Fig. 7C). Subsequently, ELISA results demonstrated that LDHA knockdown inhibited glucose accumulation and promoted pyruvate enrichment. The addition of exogenous lactate reversed this process (Fig. 7D,E). Finally, compared to the si-NC group, the ROS levels in C8-D1A and N9 cells were significantly increased in the si-LDHA group. Lactate treatment significantly reduced ROS accumulation in both C8-D1A and N9 cells (Fig. 7F). These findings demonstrate that exogenous lactate can rescue the metabolic and viability deficits caused by impaired endogenous lactate production, highlighting a direct protective role for the lactate molecule itself.

## Discussion

This study demonstrates that lactate has a remarkable neuroprotective effect in a mouse model of PSCI, a condition characterized by cognitive decline following sepsis. In the CLP-induced septic mice, exogenous lactate can improve the survival rate, body weight, and cognitive performance compared with the control group. Further analysis revealed that lactate reduces sepsis-related brain injury, a benefit that coincides with enhanced brain energy metabolism, inhibiting HMGB1/RAGE-mediated inflammatory axis, reducing oxidative stress, suppressing glial cell activation, decreasing neuronal apoptosis, and modulating the expression of specific neurotransmitter receptors, including N-methyl-D-aspartate and γ-aminobutyric acid receptors.

A key finding of our study is the potential role of lactate in influencing brain energy homeostasis. We observed that lactate upregulated the expression of GLUT1 in the hippocampal tissue of septic mice, and it increased glucose levels and reduced the harmful pyruvate accumulation. This metabolic adjustment to the disrupted environment may provide energy substrates that contribute to neuronal survival and function under the high-stress conditions of sepsis. While cognitive processes are energy-dependent, whether these metabolic changes directly mediate the observed cognitive gains requires further investigation.

Meanwhile, lactate administration effectively inhibits neuroinflammation, which is a key factor in PSCI. Notably, suppression of the HMGB1/RAGE signaling axis appears to be a key mechanism. HMGB1 is a key damage-related molecule released during sepsis that can activate RAGE on glial cells and neurons. This activation continues inflammatory responses [33, 34, 35, 36, 37]. By inhibiting the HMGB1/RAGE signaling axis, lactate inhibits a critical signaling node in sepsis-induced neuroinflammation. Consequently, the reduced activation of microglia and astrocytes and the change in cytokines from pro-inflammatory (IL-1β, IL-6, TNF-α) to anti-inflammatory (IL-10, IL-4, TGF-β) support this mechanism. The reduction of intracellular Ca^2+^ in microglia suggests improved cellular homeostasis, which may inhibit inflammatory output. By reducing this inflammatory burden, lactate creates a permissive microenvironment for neuronal function and survival, which is a prerequisite for cognitive recovery. Overall, our results support the growing idea that regulating peripheral and central inflammatory responses is crucial for cognitive recovery in PSCI.

The antioxidant effects of lactate constitute another key protective mechanism. Sepsis induces intense oxidative stress, leading to lipid peroxidation in the body, as indicated by increased MDA levels, and depletion of endogenous antioxidants such as SOD [38, 39, 40, 41]. Lactate treatment effectively reversed these effects by reducing MDA levels and increasing SOD activity. This reduction in oxidative damage is closely associated with the observed improvements in neuronal apoptosis and injury in histopathological analyses. Previous studies have demonstrated a correlation between reduced peripheral metabolic and oxidative stress markers and improved cognitive performance [42, 43, 44, 45]. Our findings extend this concept, suggesting that the antioxidant effects of lactate in both peripheral tissues and the brain may directly contribute to its cognitive benefits. Furthermore, lactate modulated the expression of key neurotransmitter receptors (GluN2B, GluA1, D1R, D2R), indicating a possible role in restoring synaptic plasticity and neuronal communication disrupted by sepsis. The restoration of these receptors, which form the molecular basis of learning and memory, provides a direct cellular link to the observed behavioral improvements. Together, the improvements in metabolism, inflammation, and oxidative stress, along with potential influences on metabolism, may create a permissive environment for synaptic remodeling, ultimately leading to enhanced cognitive outcomes. The LDHA knockdown experiments presented robust causal evidence for the protective role of lactate in PSCI. As a key rate-limiting enzyme in lactate production, LDHA knockdown directly disrupted cellular energy metabolism and redox homeostasis, leading to decreased cell viability and ROS accumulation. The subsequent supplementation with exogenous lactate significantly reversed these detrimental effects, powerfully demonstrating that lactate itself is the critical molecule mediating cellular protection, rather than acting solely as a metabolic by-product [42, 45]. These findings directly validate lactate’s pivotal role in regulating cellular energy supply and alleviating oxidative damage, providing direct molecular mechanistic support for observations in our *in vivo* and other *in vitro* models. Ultimately, this cell-level causal validation underpins how lactate’s multifaceted actions translate into the overall cognitive recovery observed in septic mice.

Some studies have considered hyperlactatemia as a marker of poor prognosis or have reported neurotoxicity of lactate under certain pathological conditions [36, 46, 47]. The inconsistency in these findings may arise from several main factors, which also represent limitations of this study. First, the effects of lactate are likely to be concentration- and time-dependent. While moderate levels or specific administration timing may exert protective effects as a metabolic substrate or signaling molecule, prolonged high-concentration exposure may exacerbate cellular acidosis or metabolic stress. Our study utilized a single dose and administration scheme; therefore, future research should systematically explore the dose range and treatment time window to determine the optimal protective effects of lactate. Second, heterogeneity of models and disease stages may influence the observed outcomes. The CLP model used in this study mainly represents acute sepsis and early cognitive impairment [36, 46]. Clinical sepsis and PSCI exhibit high variability and dynamic progression. Differences in animal models, severity of injury, and observation time points across studies may lead to varying conclusions about how lactate functions in these contexts. Furthermore, this study was limited to a single sex (male mice) [48, 49, 50, 51], did not strictly control disease severity in experimental mice, nor fully exclude interference from overall physical status (e.g., motor ability or physical strength) on cognitive function evaluation. Future studies should optimize experimental design by including both sexes to investigate potential sex-specific effects, standardizing disease severity scoring, matching physical strength across groups, and adding a control group with physical strength improvement alone. Third, complexity of mechanisms warrants further clarification. Lactate can act through different receptors (e.g., GPR81) and metabolic pathways, and its roles may differ or even oppose each other in the systemic and central nervous systems, as well as among different cell types [49, 50, 51, 52, 53]. Although this study observed an overall protective effect, the exact mechanisms by which lactate affects other molecules and inhibits the HMGB1/RAGE axis have not been fully elucidated. Moreover, this study only detected the expression levels of key molecules in the HMGB1/RAGE pathway without performing functional verification experiments such as pathway activation or inhibition, which prevents us from confirming that the HMGB1/RAGE pathway is a necessary link for lactate to exert its neuroprotective and cognitive-improving effects. This lack of clarity might explain some of the discrepancies observed in previous research results.

## Conclusions

Our results elucidate the previously overlooked role of lactate as a neuroprotective metabolite against PSCI in a mouse model. It potentially influences brain glucose metabolism, reducing oxidative stress, and suppressing neuroinflammation in a broad manner. Together, these actions protect the structure and function of neurons, ultimately rescuing cognitive abilities. These findings challenge the traditional view of lactate as merely a metabolic waste product and reframe it as a potential therapeutic option. Building on these findings, future studies will investigate the therapeutic potential of lactate to prevent or address cognitive decline in sepsis survivors, while further exploring the specific role of metabolic pathways in mediating these effects.

## Availability of Data and Materials

All the data supporting the results were shown in the paper, and can be obtained from the corresponding author.
