# Correction: Effects of Lactate on Improving Cognitive Function and Survival Rate in a Mouse Model of Post-Sepsis Cognitive Impairment

**DOI:** 10.62641/aep.v54i3.2303

**Published:** 2026-06-15

**Authors:** Jinyong Huang, Haiyong Liu, Yongwei Wu, Xiaochun Yuan, Yongtao Gao

**Affiliations:** ^1^Department of Anesthesiology, Affiliated Hospital of Nantong University, 226001 Nantong, Jiangsu, China; ^2^Medical College of Nantong University, 226001 Nantong, Jiangsu, China; ^3^Department of Anesthesiology, Yancheng City Dafeng People’s Hospital, 224100 Yancheng, Jiangsu, China; ^4^Department of Critical Care Medicine, Yancheng City Dafeng People’s Hospital, 224100 Yancheng, Jiangsu, China

The article titled “Effects of Lactate on Improving Cognitive Function and Survival Rate in a Mouse Model of Post-Sepsis Cognitive Impairment” was published in Actas Españolas de Psiquiatría, Volume 54, Issue 2, pages 301–316. In the published version of this article, errors were identified in Fig. 5.

**Fig. 5.**
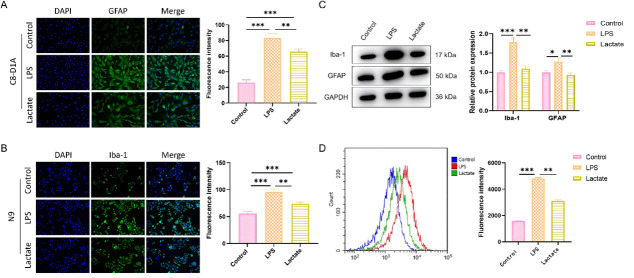
**Lactate attenuated the activation of glial cells induced by LPS**. (A) Immunofluorescence staining for GFAP in C8-D1A cells. (B) Immunofluorescence staining for Iba-1 in N9 cells. (C) Western blotting analysis of GFAP and Iba-1 protein expressions. (D) Flow cytometric assay for intracellular Ca^2+^ concentration. Data are presented as the mean ± SEM (n = 3 for each group). *p < 0.05, **p < 0.01, ***p < 0.001. Scale bars: 50 μm (A,B). LPS, lipopolysaccharide; GFAP, glial fibrillary acidic protein; Iba-1, ionized calcium-binding adaptor molecule 1.

The error in Fig. 5B occurred because the label of GFAP was written incorrectly, which should be Iba-1. The error in Fig. 5C occurred because the blot for GFAP was inadvertently omitted during the assembly of figures. All these changes do not affect the results or conclusions of this article. The authors apologize for any inconvenience caused.

The correct version of Fig. 5 is presented below.

